# Factors that facilitate or hinder the use of the facial rehabilitation webtool MEPP 2.0: a comparative study in the Quebecer health system

**DOI:** 10.1186/s12913-024-11628-2

**Published:** 2024-10-18

**Authors:** Sarah Martineau, Jacinthe Barbeau, Alyssia Paquin, Karine Marcotte

**Affiliations:** 1 Centre de Recherche de l’Hôpital Maisonneuve-Rosemont, Hôpital Maisonneuve- Rosemont, Centre Intégré Universitaire de Santé et Services Sociaux de l’Est-de-l’île- de-Montréal, Montréal, Québec Canada; 2 Centre de réadaptation Lucie-Bruneau, Centre intégré universitaire de santé et services sociaux Centre-Sud-de-l’île-de-Montréal, Montréal, Québec Canada; 3 Centre de Recherche du Centre intégré universitaire de santé et services sociaux du Nord-de-l’île-de-Montréal, Hôpital du Sacré-Coeur de Montréal, Montréal, Québec Canada; 4 Hôpital Cité-de-la Santé, Laval, Québec Canada; 5 Faculté de Médecine, Université de Montréal, Montréal, Québec Canada; 6 Maisonneuve-Rosemont Hospital, 5415 boul. De l’Assomption, Montréal, H1T 2M4 Canada

**Keywords:** Facial rehabilitation, Mirror therapy, Peripheral facial palsy, Technology, E-health, MEPP

## Abstract

**Background:**

Recently, our research team developed an open source and free website called the MEPP website (for the Mirror Effect Plus Protocol) to efficiently provide mirror therapy for patients with facial palsy. Previous studies demonstrated that the first version of the MEPP website improved user experience and likely optimized patients’ performance during facial therapy. Nevertheless, compliance was found to be low despite a generally positive opinion of the website, and in light of our earlier findings, MEPP 2.0—a revised and enhanced version of the MEPP 0.1—was created. The purpose of this study was to examine and contrast various factors that help or impede institutional partners of the Quebec health care system from using the MEPP 2.0 website in comparison to its initial version.

**Methods:**

Forty-one patients with facial palsy and nineteen clinicians working with this population were enrolled in a within-subject crossover study. For both the MEPP 1.0 and MEPP 2.0, user experience was assessed for all participants. Embodiment was assessed in patients, and factors influencing clinical use were assessed by clinicians. Qualitative comments about their experiences were also gathered. Descriptive statistics and reliability measures were calculated. Differences between the two MEPP versions were assessed using the linear mixed model.

**Results:**

Overall, patients appreciated more the MEPP 2.0 (OR = 4.57; *p* < 0.001), and all clinicians preferred the MEPP 2.0 over the MEPP 1.0. For patients, it seems that facial ownership, as well as possession and control of facial movements, was significantly better with the MEPP 2.0. For clinicians, the MEPP 2.0 specifically allowed them to self-evaluate their intervention and follow up with more objectivity. The use of the MEPP 2.0 was also modulated by what their patients reported. Qualitatively, options to access an Android app and needs for improving the exercises bank were mentioned as hindering factors.

**Conclusions:**

The updated version of the MEPP website, the MEPP 2.0, was preferred by our different partners.

**Trial registration:**

https://www.isrctn.com/ISRCTN10885397. The trial was registered before the start of the study on the 1^st^ December 2023

**Supplementary Information:**

The online version contains supplementary material available at 10.1186/s12913-024-11628-2.

## Background

In recent years, the use of technology has shown great potential in health and rehabilitation services. Specifically, the use of new technologies has demonstrated many advantages, such as improved accessibility and efficient and effective care for patients, thereby enhancing rehabilitation [22, 25]. However, technology resistance and abandonment have also been reported with both users and health service providers in the rehabilitation realm [15]. The literature indicates that technological challenges stem from stakeholder mismatches: users (people with disabilities) and their priorities, health service providers and their preferences, and technology designers/manufacturers and their realities [15, 25]. Therefore, a more user-centered approach is recommended for the development of this technology, especially by considering the perspectives of users and health service providers in real-world settings to address these barriers [15].

### Development of MEPP technology for facial rehabilitation

**Fig. 1:**
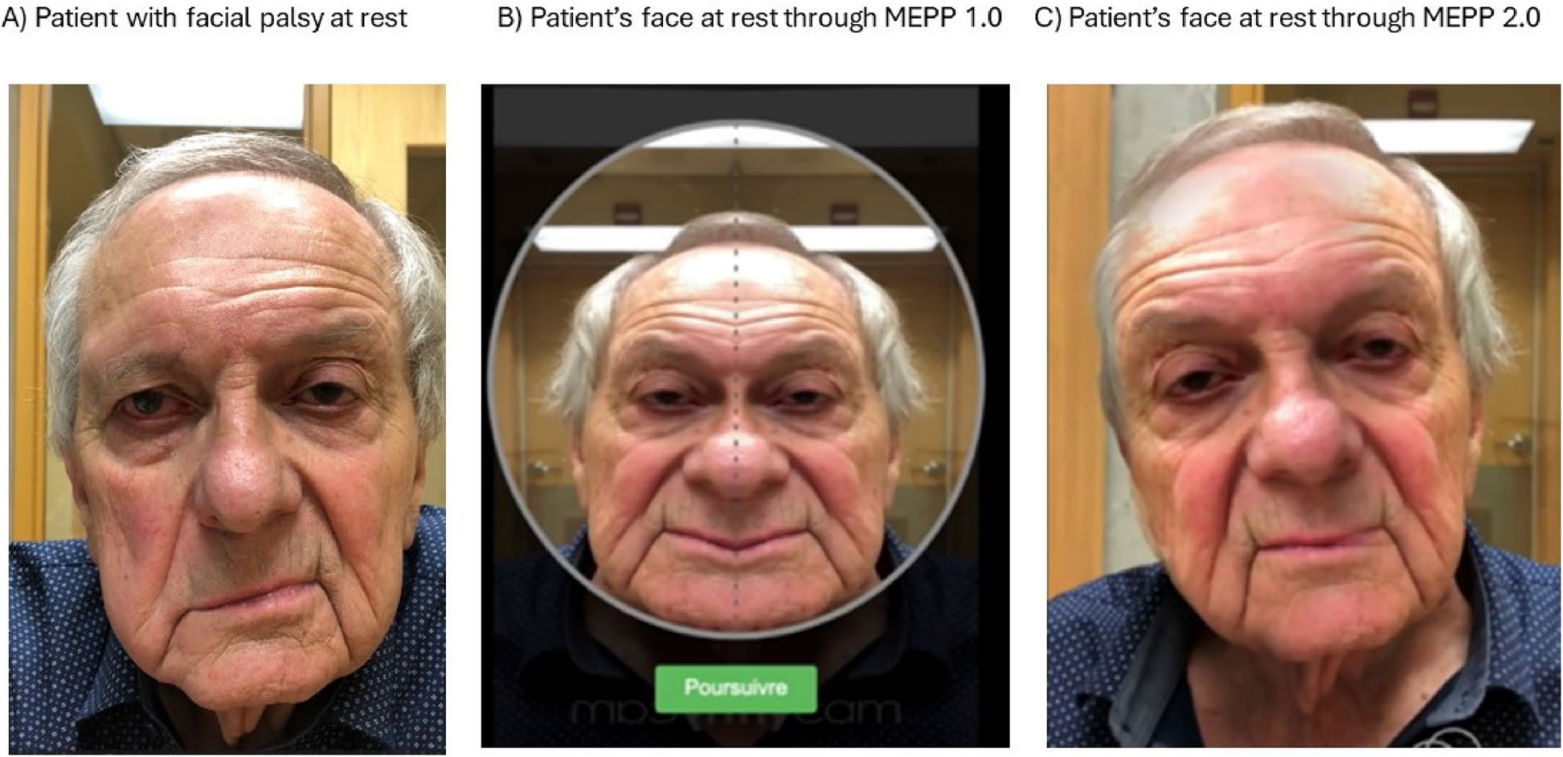
Patient’s face at rest (**A**) with Comparisons of facial mirroring between (**B**) the MEPP-1.0 and (**C**) the MEPP-2.0

To address the absence of tailored facial rehabilitation services for patients in Quebec, the MEPP 1.0, a web-based facial rehabilitation tool, was created in 2020 [10]. This web-based technology was designed considering that the use of digital technology has been proven to be effective in treating facial palsy [22, 23]. Moreover, MEPP 1.0 has been demonstrated to possess intrinsic characteristics that encourage the use of technology in rehabilitation, such as being a readily available and usable technology, demonstrating favorable functional outcomes, and being a reliable and credible technology [11, 15, 25]. MEPP 1.0 is based on the principle of mirror therapy [20], meaning that it employs modified visual feedback during facial retraining to create the illusion that the face is moving symmetrically. This is achieved by mirroring the healthy side onto the paralyzed side, thus promoting normalized motor execution [9]. However, despite a generally high appreciation of the website, compliance with therapy was found to be relatively low. Based on these results, a new and improved version of the MEPP was developed [10]. Like the first version, the MEPP 2.0 is available freely. However, it uses augmented reality for better facial rendering during mirror therapy (see Fig. 1). Moreover, enhanced security, compartmentalization of the patients’ and clinicians’ information, and the possibility of providing an overview of the patients’ profile from the clinician’s account constitute the main improvements of the second version. It is also available through an app for Apple mobile devices, which was not the case for the MEPP 1.0. Since no other tool is available in French or English, it has gained appreciation locally and internationally, with more than 350 users already using it. However, the factors modulating the use of this tool in clinical settings are still relatively unknown and must be studied empirically to enhance rehabilitation and avoid abandonment.

### Factors hindering or facilitating technology usage in rehabilitation

Different factors seem to support the future use of rehabilitation technologies. Among them, assessing the user experience during the use of a new technology is an important factor [6, 13, 24]. Moreover, in the context of mirror therapy for facial palsy rehabilitation, assessing factors that potentially affect self-identification with the facial image, such as the mirroring effect in the self or the patient’s appreciation of embodiment, is essential [3, 12, 21]. Finally, considering all stakeholders using the technological tool during its development, including clinicians, is also imperative [15, 16, 25].

#### User experience

User experience is a concept that allows assessing the interactions between a user and its technological tool. Thüring and Mahlke [24] developed a user experience analytical model that assesses different components: the perception of instrumental qualities (such as utility and usability) and non instrumental qualities (such as aesthetics) of the technology, the influence of the user’s emotions regarding the tool, the consequences of the components of loyalty and intent toward the tool and the global assessment of the tool [14]. The assessment of user experience allows us to consider users’ opinions on technology quality and performance while preventing technology abandonment.

#### Augmented reality and embodiment

A frequent complaint of the MEPP 1.0 was the instability of the mirroring effect by the central axis. This instability of the mirroring effect can make the face appear less natural, which affects embodiment [3]. The embodiment concept is described by the capacity to identify an image of ourselves and associate it with our proprioception [12]. Therefore, to remedy the problem of instability of the mirroring effect in the MEPP 1.0, the MEPP 2.0 uses augmented reality technology for the mirroring effect. This technology enables a more realistic and stable image, which has also been confirmed in similar studies [3, 4]. Moreover, augmented reality also reduces the interruptions and extrinsic complexity of therapy linked with the face’s position, which in turn reduces the task’s cognitive load, assuring a better user experience and motor learning [5, 17].

#### Clinical factors influencing practice

As mentioned previously, when analyzing the use of a rehabilitation technology, it is important to consider all stakeholders using the technological tool [15]. Clinicians are important stakeholders as they play a significant role in using and adapting the MEPP to patients’ specific context and situation. The clinician, therefore, also represents a user of the technological tool but with a clinical perspective. Factors modulating the use of the MEPP by clinicians not only include their user experience but also their clinical practice [15, 16]. Thus, characterizing the clinical factors influencing their practice is important.

Moreover, self-assessment is an essential aspect of clinical practice, as it enables clinicians to assess their own skills and determine their strengths and weaknesses to develop and maintain competence [7, 16]. It ensures quality care and clinical excellence. Employing clinician self-assessment allowed us to consider the clinical factors affecting the use of the MEPP tool.

### Objectives

The aim of this study was to measure and compare different factors that could facilitate or hinder the use of the MEPP 2.0 website compared to its first version. More specifically, we aimed to compare the experiences of patients and clinicians, the virtual facial embodiment of patients and factors influencing the clinical practice of clinicians. We hypothesised that the MEPP 2.0 would allow all stakeholders to benefit from a better user experience, that the facial embodiment would be better with the MEPP 2.0 but that clinical factors influencing practise wouldn’t differ in both tools.

## Methods

This prospective, multicentric randomized control trial was approved by the Ethics Committee of the *Centre de Recherche du Centre intégré universitaire de Santé et Services Sociaux de l’Est-de-l’île-de-Montréal* (MP-12-2023-3218). The data analyzed in this study were gathered following the CONSORT guidelines for transparent controlled trials (registered on the 1st December 2023 at *ISRCTN10885397*). Written, free, and informed consent to participate was obtained from every participant prior to study enrollment.

### Participants

#### Patients

Fifthy-one patients with facial palsy who came from various health establishments in Quebec were assessed for eligibilty, and forty-one (41) (CIUSSS de l’Est-de-l’Île-de-Montréal, CIUSSS Centre-Sud-de-l’Île-de-Montréal, CHUM, CIUSSS de la Capitale Nationale, CISSS de la Côte-Nord, and CISSS de la Montérégie-Ouest) participated in this study. Patients for whom a MEPP username was registered were preselected and contacted by their treating clinicians to obtain pre-consent to be contacted by the research team. Patients who pre-consented were then contacted by a research team member to ensure that they met the inclusion criteria, and provided written consent. Patients were recruited according to the following inclusion criteria: (1) were aged 18 years or older, (2) had peripheral facial paralysis, (3) had used or agreed to use the MEPP 1.0 and the MEPP 2.0, and (4) spoke and read French and/or English sufficiently to be able to complete an oral interview and standardized questionnaires. The exclusion criteria were (1) having a major cognitive disorder hindering the ability to answer questionnaires and interview questions for this project. All patients were randomized before starting any study procedure (see below).

#### Clinicians

Twenty-eight (28) clinicians working with clients suffering from facial paralysis were assessed for eligibility, and nineteen (19) of them, who came from various health establishments in Quebec (CIUSSS de l’Est-de-l’Île-de-Montréal, CIUSSS Centre-Sud-de-l’Île-de-Montréal, CIUSSS de la Capitale Nationale, CISSS de Chaudière-Appalaches, CISSS de Laval, and CISSS de la Montérégie-Ouest) participated in this study. They were contacted by the research team via email or telephone to probe interest in their participation in the study because they were users of the MEPP website. After initial interest was confirmed, the clinicians were recruited according to the following inclusion criteria: (1) aged 18 years or older, (2) used or agreed to use the MEPP 1.0 and MEPP 2.0 in the clinic or during a standardized trial allowing informed comparative impressions to be obtained on both tools, and (3) spoke and read French and/or English sufficiently to be able to complete an interview and standardized questionnaires. There were no exclusion criteria for clinician participation in this study. All clinicians were randomized before starting any study procedure (see below).

### Study design

**Fig. 2:**
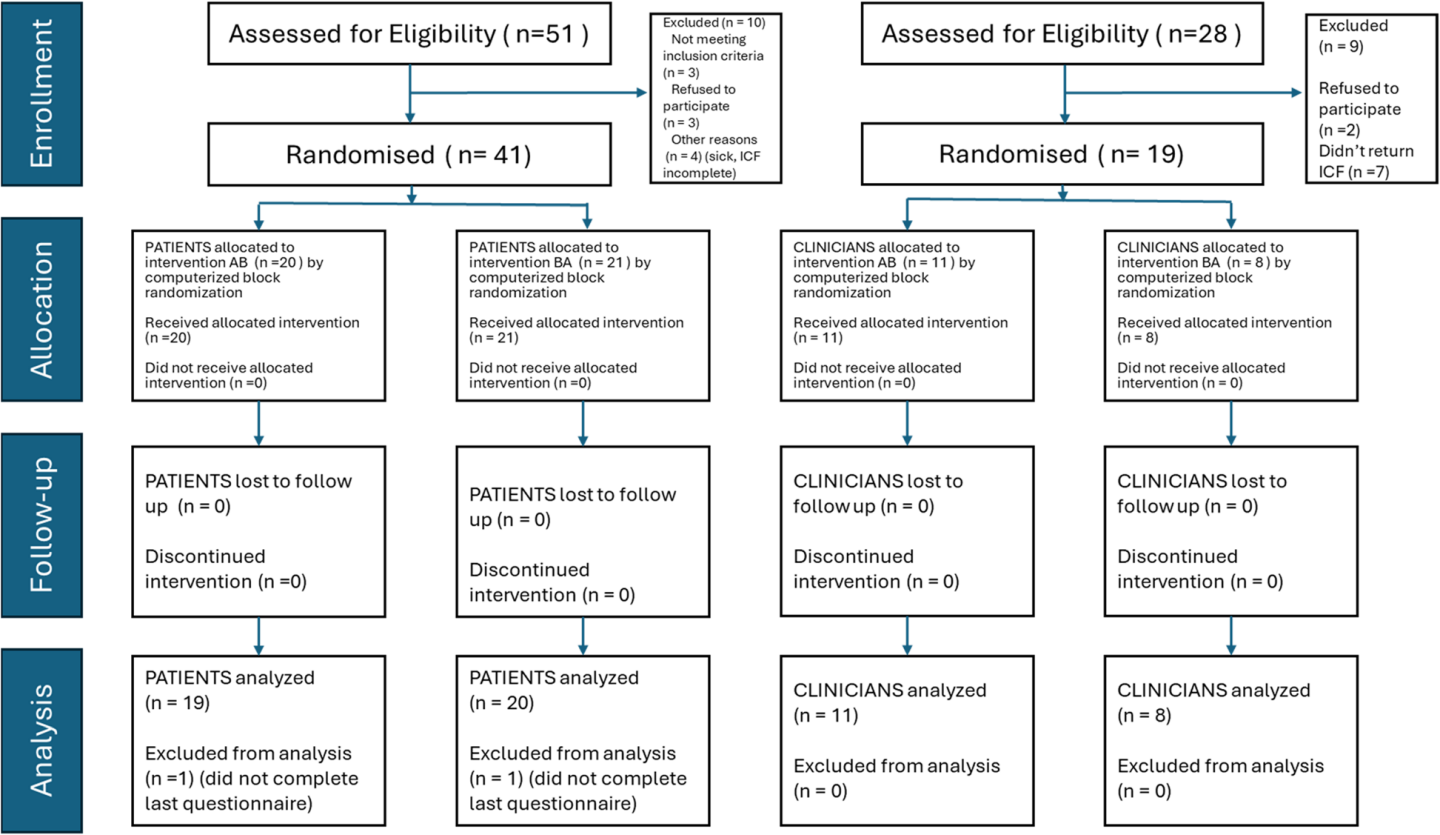
CONSORT Study Flowchart. Legend: BA = MEPP 2.0 (Blue presented first and MEPP 1.0 (Green) presented second

Figure 2 shows the study flowchart. There were two groups for each sequence, one for patients and one for clinicians. Participants were assigned to one of the following groups via computerized block randomization: group AB started with the MEPP 1.0, and group BA started with the MEPP 2.0. Throughout this study, when talking with all participants, we used the terms “green MEPP” for the MEPP 1.0 and “blue MEPP” for the MEPP 2.0. These terms were chosen according to the visual aspects of each tool to avoid bias. Each participant took part in a standardized trial lasting a minimum of two weeks. Patients were advised to carry out the intervention plan programmed for their personal needs, according to the facial impairments to address, as assessed by the clinicians, at a minimum of one to two sessions per day. The intervention plans generally lasted five to fifteen minutes per session (10 to 30 min of practice distributed throughout the day). Clinicians were asked to explore each website (green and blue MEPP) using training material designed for it. They were asked to set up an intervention plan, explain the functioning of the website, and adjust the plan to the patient. If no patient was readily available at the time of the study, clinicians manipulated the web tools with a fictitious patient. At the end of each standardized trial (one for the green MEPP-1.0 and one for the blue MEPP 2.0), each participant was met by a member of the research team to carry out a telephone or virtual interview, during which standardized questionnaires were completed to assess user experience, virtual embodiment and self-assessed internal/external factors influencing clinical use of the websites depending on what was relevant. Qualitative impressions and open comments were also collected during those interviews.

#### Outcome measures

##### MeCUE questionnaire

The MeCUE 2.0 (Modular Evaluation of Components of User Experience; http://mecue.de/*)* is a standardized questionnaire aimed at measuring the concept of user experience. This questionnaire, which was translated into French by Lallemand and Koenig [6], is adaptable, complete and valid [13, 14]. It is composed of 4 key components: perception of the product, emotions, consequences of use and overall evaluation. MeCUE scores are calculated through 10 subsectional scales: usefulness, usability, loyalty, aesthetics, status, commitment, negative emotions, positive emotions, usage intention and overall evaluation. The MeCUE [13] can be found in the supplementary material 1.

##### VEQ

The Virtual Embodiment Questionnaire (VEQ -Roth & Latoschik [21], is a questionnaire specific to virtual body identification in augmented reality. Since the blue MEPP 2.0 uses augmented reality for facial mirroring, which involves a new technology for virtual body identification, the VEQ was used to assess this aspect. The questionnaire has 3 components, each composed of 4 questions: body ownership (corresponds to “possession of the virtual body”), agency (or “control of one’s own movements”) and change in perceived body scheme (or “perceived changes”) [21]. In the present study, the second component of the VEQ is especially relevant because it is specific to changes in the virtual face’s appearance and measurements based on the subject’s movements. It should be noted that this scale shows great sensitivity to the control of one’s own movements. Finally, each component is scored individually, which makes the questionnaire very adaptable [21]. The VEQ (VEQ -Roth & Latoschik [21]), can be found in the supplementary material 2.

#### Clinical factors

The clinical factors influencing the practices of clinicians were assessed using a questionnaire based on Orest work, which includes a self-assessment perspective of clinical practice. Two main process categories are emphasized in this questionnaire: the internal processes of the clinician and the external processes of the clinician. In addition, the structure of the interviews allowed us to categorize internal processes in terms of motivators for use or barriers to use, and to compare the differences between these processes for the two applications, namely, green MEPP 1.0 and blue MEPP 2.0 [16]. The structure of the interview based on Orest [16] can be found in the supplementary material 3.

##### Overall preference

The question “Which of the MEPP versions did you prefer?” was asked once at the end of both trials, and patients had to respond with blue or green.

##### Qualitative impressions

Subjective impressions were gathered informally from both patients and clinicians at the end of their respective standardized trials for each website. They were asked to provide suggestions for improvements, discuss factors that influenced the use of a software platform over the other and express their preference for a website as well as reasons why.

#### Data analysis

All the statistical analyses were performed using R version 4.1.2 Team Core [1]. Descriptive data were generated and verified for normality with the Shapiro‒Wilk test. The reliability of the measures was calculated for the MeCUE with Cronbach’s alpha. Linear mixed-effect (lme) models were adjusted to test for differences in the two versions of the different variables (user experience, virtual embodiment and clinical factors) while checking for a crossover effect. The fixed effects are the version (green MEPP 1.0 vs. blue MEPP 2.0), the order of the presented version (AB vs. BA, meaning respectively, MEPP 1.0 presented first vs. MEPP 2.0 presented first) and, finally, the crossover effect (version order interaction). Relevant post hoc contrasts were calculated depending on the situation (order was taken into account when it had an effect or not when no effect was detected). For overall preference, odd ratios and chi-square tests were performed. All tests were conducted with a*p* value of 0.05.

## Results

### Participants’ demographics

Participants’ demographic information is reported in Table 1. Among patients, two had not completed both questionnaires for the website versions and were consequently excluded from the analyses. Thus, 39 patients [females, 22; mean (sd) age: 49.5 (14.5) y.o.; schooling < 12 years: n (%) 24 (61.5%); French language 31 (79.5%); left-sided facial palsy 17 (43.6%); diagnosis of Bell’s palsy 20 (52.6%)] and 19 clinicians [females, 18; mean (sd) age, 33.8 (8.9); years of experience in field 9.1 (9.3); training in FP, 9 (47.4%)] were enrolled.

**Table 1:** Demographic information of the patients and clinicians

Patients (n = 41)		Clinicians (n = 19)	
Females n=22	Males n=19	Females n=18	Males n=1
Variables	N (%) / Mean (sd)	Variables	N (%) / Mean (sd)
Age	49.5 (14.5)	Age	33.8 (8.9)
Schooling		Language	
<12 years	24 (61.5%)	French	19 (100%)
> 12 years	15 (38.5%)	Years of practise	9.1 (9.3)
Language			
French	31 (79.5%)		
English	4 (10.3%)		
Spanish	1 (2.6%)		
Other	3 (7.7%)		
Side of FP			
Left	17 (43.6%)		
Right	22 (56.4%)		
Diagnostic			
Bell’s Palsy	20 (52.6%)		
Ramsay-Hunt	5 (13.2%)		
Acoustic neurinoma	5 (13.2%)		
Traumatic injury	3 (7.9%)		
Other	5 (13.2%)		

### User experience

Descriptive statistics are presented in supplementary material 4. In patients, as shown in Table 2, the variables usefulness (F = 10.37; *p* = 0.003), status (F = 4.6; *p* = 0.040), positive (F = 7.3; *p* = 0.010) and negative (F = 4.6; *p* = 0.038) emotions, intention to use (F = 6.0; *p* = 0.020), and overall (F = 5.5; *p* = 0.025) demonstrated a crossover effect. This means that the differences between the preferred versions depended on the order of presentation. Therefore, post hoc contrasts between versions were performed by taking the order into account (see Table 3). Overall, version 2 was much more appreciated, but for all subscales with crossover effects, version 2 was mostly preferred when it was presented first.

**Table 2:** Anova measuring Impact of Version (MEPP 1.0 vs MEPP 2.0) while checking for Crossover Effect (Version Order) in Patients

	I - Usefulness			
	df1	df2	F	*p*-value
**Version**	**1**	**37**	**12.3**	**0.0012**
Order	1	37	0.85	0.36
**Version** **Order**	**1**	**37**	**10.37**	**0.003**
	I - Usability			
	df1	df2	F	*p*-value
**Version**	**1**	**37**	**52.9**	**<0.001**
Order	1	39	0.1	0.77
Version Order	1	37	1.2	0.28
	II - Visual Aesthetic			
	df1	df2	F	*p*-value
**Version**	**1**	**37**	**29.4**	**<0.001**
Order	1	39	0.4	0.51
Version Order	1	37	0.0	0.95
	II - Status			
	df1	df2	F	*p*-value
**Version**	**1**	**32**	**4.2**	**0.049**
Order	1	36	0.3	0.62
**Version** ** Order**	**1**	**32**	**4.6**	**0.040**
	II - Commitment			
	df1	df2	F	*p*-value
**Version**	**1**	**37**	**10.9**	**0.002**
Order	1	39	2.1	0.15
Version Order	1	37	2.9	0.10
	III - Positive emotions			
	df1	df2	F	*p*-value
**Version**	**1**	**37**	**12.6**	**0.001**
Order	1	39	0.1	0.71
**Version** ** Order**	**1**	**37**	**7.3**	**0.010**
	III - Negative emotions			
	df1	df2	F	*p*-value
**Version**	**1**	**37**	**23.8**	**<0.001**
Order	1	39	0.1	0.73
**Version** ** Order**	**1**	**37**	**4.6**	**0.038**
	IV - Intention to use			
	df1	df2	F	*p*-value
**Version**	**1**	**36**	**9.0**	**0.005**
Order	1	39	0.3	0.57
**Version** ** Order**	**1**	**36**	**6.0**	**0.020**
	IV – Loyalty			
	df1	df2	F	*p*-value
**Version**	**1**	**35**	**37.6**	**<0.001**
Order	1	39	1.4	0.25
**Version** ** Order**	**1**	**35**	**7.8**	**0.008**
	V - Overall			
	df1	df2	F	*p*-value
**Version**	**1**	**36**	**23.6**	**<0.001**
Order	1	39	0.1	0.80
**Version Order**	**1**	**36**	**5.5**	**0.025**

*Legend*: bold = statistically significant results; df = degrees of freedom; F = ANOVA

**Table 3:** Marginal expected means and post-hoc contrasts for the Linear mixed-effect models on MeCUE subscales in patients

MeCUE subscales	Order	Mean (SE) Version Green-V1.0	Mean (SE) Version Blue-V2.0	t	*p*-value
I - Usefulness	AB	5.10 (0.30)	5.18 (0.29)	0.26	0.80
	**BA**	**4.64 (0.30)**	**6.23 (0.29)**	**4.75**	**< 0.001**
I - Usability	**AB**	**4.85 (0.27)**	**6.44 (0.26)**	**4.45**	**< 0.001**
	**BA**	**4.65 (0.27)**	**6.80 (0.26)**	**5.86**	**< 0.001**
II – Visual Aesthetic	**AB**	**4.48 (0.27)**	**5.79 (0.27)**	**3.93**	**< 0.001**
	**BA**	**4.30 (0.27)**	**5.57 (0.27)**	**3.74**	**< 0.001**
II - Status	AB	3.88 (0.32)	3.86 (0.29)	-0.05	0.96
	**BA**	**3.08 (0.32)**	**4.23 (0.29)**	**2.95**	**0.006**
II - Commitment	AB	2.57 (0.32)	3.00 (0.32)	1.17	0.25
	**BA**	**2.66 (0.32)**	**3.97 (0.32)**	**3.52**	**0.001**
III - Positive Emotions	AB	3.78 (0.27)	3.98 (0.27)	0.65	0.52
	**BA**	**3.05 (0.27)**	**4.44 (0.27)**	**4.42**	**< 0.001**
III - Negative Emotions	AB	2.81 (0.25)	2.28 (0.24)	-1.99	0.05
	**BA**	**3.34 (0.25)**	**1.98 (0.24)**	**-4.94**	**< 0.001**
IV – Intention to use	AB	3.77 (0.31)	3.94 (0.30)	0.47	0.64
	**BA**	**3.32 (0.31)**	**4.73 (0.30)**	**3.83**	**< 0.001**
IV - Loyalty	**AB**	**3.74 (0.32)**	**4.83 (0.31)**	**2.44**	**0.020**
	**BA**	**2.43 (0.32)**	**5.29 (0.31)**	**6.29**	**< 0.001**
V - Overall	AB	1.69 (0.50)	2.98 (0.49)	1.88	0.07
	**BA**	**0.36 (0.50)**	**3.95 (0.49)**	**5.06**	**< 0.001**

Legend: bold = statistically significant results; *SE* standard error, *AB* green MEPP 1.0 presented first and blue MEPP 2.0 presented second; BA = blue MEPP 2.0 presented first and green MEPP Green 1.0 presented second

Table 4 shows that for clinicians, the variables usefulness (F = 7.2; *p* = 0.016), negative emotions (F = 6.0; *p* = 0.026), and overall appreciation (F = 9.5; *p* = 0.007) demonstrated a crossover effect. This means that the differences between the versions for these concepts depended on the order of presentation. Therefore, post hoc contrasts for differences between preferred versions were performed by taking the order into account. This is shown in Table 5. Overall, the blue MEPP 2.0, was much more appreciated. There were significantly more negative emotions associated with green MEPP 1.0, when the blue MEPP-2.0 was presented first. Users had significantly greater intentions to use the blue MEPP 2.0 when it was presented first. There are no differences in commitment, and for all other variables, version 2.0 is preferred in both order.

**Table 4:** Anova Measuring Impact of Version while checking for crossover effect (Version $\:\times\:$ Order) in clinicians

	I - Usefulness			
	df1	df2	F	*p*-value
**Version**	**1**	**17**	**36.8**	**< 0.001**
Order	1	17	2.1	0.17
**Version** $\:\times\:$ **Order**	**1**	**17**	**7.2**	**0.016**
	I - Usability			
	df1	df2	F	*p*-value
**Version**	**1**	**17**	**14.5**	**0.001**
Order	1	17	0.7	0.42
Version$\:\times\:$Order	1	17	0.7	0.40
	II - Visual Aesthetic			
	df1	df2	F	*p*-value
**Version**	**1**	**17**	**41.1**	**< 0.001**
Order	1	17	5.4	0.033
Version$\:\times\:$Order	1	17	0.3	0.59
	II - Status			
	df1	df2	F	*p*-value
**Version**	**1**	**17**	**43.2**	**< 0.001**
Order	1	17	1.9	0.18
Version$\:\times\:$Order	1	17	4.3	0.05
	II - Commitment			
	df1	df2	F	*p*-value
Version	1	17	4.3	0.05
Order	1	17	3.9	0.07
Version$\:\times\:$Order	1	17	0.3	0.56
	III - Positive emotions			
	df1	df2	F	*p*-value
**Version**	**1**	**17**	**18.7**	**< 0.001**
Order	1	17	0.5	0.50
**Version** $\:\times\:$ **Order**	1	17	3.0	0.10
	III - Negative emotions			
	df1	df2	F	*p*-value
**Version**	**1**	**17**	**12.6**	**0.002**
Order	1	17	1.3	0.27
**Version** $\:\times\:$ **Order**	**1**	**17**	**6.0**	**0.026**
	IV - Intention to use			
	df1	df2	F	*p*-value
**Version**	**1**	**17**	**12.2**	**0.003**
Order	1	17	2.7	0.12
Version$\:\times\:$Order	1	17	1.5	0.23
	IV – Loyalty			
	df1	df2	F	*p*-value
**Version**	**1**	**17**	**91.9**	**< 0.001**
**Order**	**1**	**17**	**11.8**	**0.003**
Version$\:\times\:$Order	1	17	1.2	0.29
	V - Overall			
	df1	df2	F	*p*-value
**Version**	**1**	**17**	**44.7**	**< 0.001**
Order	1	17	8.8	0.009
**Version** $\:\times\:$ **Order**	**1**	**17**	**9.5**	**0.007**

Legend: bold = statistically significant results; df = degrees of freedom; F = ANOVA

**Table 5:** Marginal expected means and post-hoc contrasts for the Linear mixed-effect models on MeCUE subscales in clinicians

MeCUE subscales	Order	Mean (SE) Version Green-1.0	Mean (SE) Version Blue-2.0	t	*p*-value
I - Usefulness	**AB**	**5.39 (0.31)**	**6.18 (0.31)**	**2.88**	**0.010**
	**BA**	**4.21 (0.31)**	**6.12 (0.31)**	**5.97**	**< 0.001**
I - Usability	**AB**	**4.52 (0.44)**	**5.85 (0.44)**	**2.35**	**0.031**
	**BA**	**3.71 (0.44)**	**5.79 (0.44)**	**3.13**	**0.006**
II – Visual aesthetic	**AB**	**4.39 (0.32)**	**6.18 (0.32)**	**5.23**	**< 0.001**
	**BA**	**3.58 (0.32)**	**5.08 (0.32)**	**3.74**	**0.002**
II - Status	**AB**	**4.21 (0.30)**	**5.21 (0.30)**	**3.66**	**0.002**
	**BA**	**3.21 (0.30)**	**5.08 (0.30)**	**5.85**	**< 0.001**
II - Commitment	AB	3.00 (0.32)	3.55 (0.32)	1.95	0.07
	BA	2.25 (0.32)	2.54 (0.32)	0.89	0.39
III – Positive emotions	**AB**	**3.62 (0.28)**	**4.17 (0.28)**	**2.18**	**0.044**
	**BA**	**3.02 (0.28)**	**4.23 (0.28)**	**4.12**	**< 0.001**
III – Negative emotions	AB	2.15 (0.29)	1.76 (0.29)	-1.11	0.28
	**BA**	**3.23 (0.29)**	**1.50 (0.29)**	**-4.16**	**< 0.001**
IV – Intention to use	AB	3.88 (0.30)	4.52 (0.30)	1.86	0.08
	**BA**	**2.92 (0.30)**	**4.21 (0.30)**	**3.21**	**0.005**
IV – Loyalty	**AB**	**3.52 (0.28)**	**5.76 (0.28)**	**6.59**	**< 0.001**
	**BA**	**2.04 (0.28)**	**4.85 (0.28)**	**7.05**	**< 0.001**
V – Overall	**AB**	**2.18 (0.49)**	**3.95 (0.49)**	**3.09**	**0.007**
	**BA**	**-1.00 (0.49)**	**3.50 (0.49)**	**6.68**	**< 0.001**

Legend: bold = statistically significant results; SE = standard error; AB = MEPP Green 1.0 presented first and MEPP Blue 2.0 presented second; BA = MEPP Blue 2.0 presented first and MEPP Green 1.0 presented second

#### Virtual embodiment

In module 1 (acceptance/body ownership), all items had a statistically significant version, but there was a small crossover effect, so post hoc tests were performed according to the order of presentation. Table 6 shows that blue MEPP 2.0 is preferred, but it is statistically significant only when presented first, except for item 4, which is statistically significant in both presentations (t = 2.13; *p* = 0.040 & t = 4.66; p = < 0.001). In module 2 (control/agency), all items had a version effect that was statistically significant or marginally significant without any crossover effect. Consequently, post hoc tests were performed to compare the versions only. The blue MEPP 2.0 was preferred, but it was statistically significant only for the following items: item 1 (possession of the movements: t = 2.70, *p* = 0.010); item 4 (synchronized movements: t = 2.21, *p* = 0.033); and total score ( t = 2.69, *p* = 0.011). In module 3 (perceived changes), all items had a version effect that was statistically significant or marginally significant without any crossover effect. Consequently, post hoc tests were performed comparing versions only, and it could be seen that the second version demonstrated fewer changes in the face and that it was statistically significant for all items except for item 1 (t= -1.44; *p* = 0.16). This means that blue MEPP 2.0 facilitates embodiment.

**Table 6:** Marginal expected means and post-hoc contrasts for the Linear mixed-effect models on virtual embodiment questionnaire (VEQ) by Version and Order of Presentation

VEQ		Order	Mean (SE) Green- V1.0	Mean (SE) Blue-V2.0	t	*p*-value
Module 1 – Acceptance/Body Ownership	Item 1 : my Body	AB	3.89 (0.42)	4.70 (0.40)	1.39	0.17
		**BA**	**2.58 (0.42)**	**5.48 (0.40)**	**5.05**	**< 0.001**
	Item 2 : my Body Parts	AB	4.17 (0.41)	4.75 (0.40)	1.09	0.28
		**BA**	**3.09 (0.41)**	**5.71 (0.40)**	**4.95**	**< 0.001**
	Item 3 : Humanness	AB	4.11 (0.39)	5.10 (0.38)	1.92	0.06
		**BA**	**3.69 (0.39)**	**6.10 (0.38)**	**4.72**	**< 0.001**
	Item 4: Belongs to me	**AB**	**3.95 (0.42)**	**5.15 (0.41)**	**2.13**	**0.040**
		**BA**	**3.27 (0.42)**	**5.86 (0.41)**	**4.66**	**< 0.001**
	Total	**AB**	4.03 (0.38)	4.92 (0.37)	1.76	0.09
		**BA**	**3.15 (0.38)**	**5.79 (0.37)**	**5.23**	**< 0.001**
Module 2 – Agency/control of movements	Item 1 : Mine	**4.74 (0.27)**		**5.65 (0.26)**	**2.70**	**0.010**
	Item 2 : Control	4.98 (0.28)		5.48 (0.28)	1.66	0.11
	Item 3: Cause	5.31 (0.24)		5.76 (0.24)	1.74	0.09
	Item 4 : Synchrony	**5.25 (0.25)**		**5.92 (0.24)**	**2.21**	**0.033**
	Total	**5.07 (0.22)**		**5.70 (0.22)**	**2.69**	**0.011**
Module 3– Perceived changes	Item 1: My Body changes	5.04 (0.32)		4.46 (0.32)	-1.44	0.16
	Item 2: Heavy Light	**4.82 (0.31)**		**3.26 (0.30)**	**-3.86**	**< 0.001**
	Item 3: Tall Small	**3.54 (0.26)**		**2.23 (0.25)**	**-4.09**	**< 0.001**
	Item 4: Large Thin	**4.87 (0.32)**		**3.30 (0.31)**	**-3.75**	**< 0.001**
	Total	**4.57 (0.25)**		**3.32 (0.24)**	**-3.91**	**< 0.001**

Legend: bold = statistically significant results; *SE* standard error, *AB *MEPP Green 1.0 presented first and MEPP Blue 2.0 presented second; BA = MEPP Blue 2.0 presented first and MEPP Green 1.0 presented second

### Clinical factors

As shown in Table 7, regarding module 1 of the questionnaire (internal processes), the blue MEPP 2.0 was preferred for items 6 (allows me to intervene with more objectivity), 7 (allows me to objectively assess progress) and 8 (allows me to gain experience). Consequently, the mean score of module 1 was also higher for blue MEPP 2.0. Since there was no crossover effect, the contrasts were conducted on the variable “version” only. The contrast between both versions was only statistically significant for items 6 (t = 3.58; *p* = 0.003), 7 (t = 2.22; *p* = 0.046) and 8 (t = 2.98; *p* = 0.008), and marginally significant for the total score. Regarding module 2 (external processes), the blue MEPP 2.0 was preferred for items 5 (continuing education) and 8 (information reported by my patient). Consequently, the mean score of module 2 was also higher for this version. Since there was no crossover effect, the contrast was performed on the variable “version” only, and the results revealed that differences between green MEPP 1.0 and blue MEPP 2.0 were only statistically significant for items 5 (continuing education; t = 2.48; *p* = 0.025) and 8 (information reported by my patient; t = 2.49; *p* = 0.024).

**Table 7:** Marginal expected means and post-hoc contrasts for the Linear mixed Effect models of Internal and external clinical factors influencing practice questionnaire by version and order of presentations for clinicians

Clinical factors		Mean (SE) green MEPP 1.0	Mean (SE) blue MEPP 2.0	t	*p*-value
Module 1 – Internal Processes	Item 1	6.52 (0.14)	6.63 (0.14)	0.61	0.55
	Item 2	3.61 (0.36)	3.06 (0.36)	-1.80	0.09
	Item 3	2.28 (0.39)	2.29 (0.39)	0.05	0.96
	Item 4	4.05 (0.42)	4.68 (0.42)	1.56	0.14
	Item 5	4.76 (0.47)	5.58 (0.46)	1.75	0.10
	**Item 6**	**4.48 (0.29)**	**5.62 (0.30)**	**3.58**	**0.003**
	**Item 7**	**4.05 (0.43)**	**5.01 (0.45)**	**2.22**	**0.046**
	**Item 8**	**4.91 (0.28)**	**5.79 (0.28)**	**2.98**	**0.008**
	Item 9	3.64 (0.45)	3.60 (0.45)	-0.10	0.92
	Item 10	2.92 (0.34)	2.82 (0.34)	-0.37	0.72
	Item 11	3.49 (0.45)	3.68 (0.45)	0.34	0.74
	Item 12	1.40 (0.18)	1.34 (0.18)	-0.31	0.76
	Item 13	2.88 (0.44)	2.22 (0.44)	-1.31	0.21
	Item 14	1.79 (0.21)	1.44 (0.21)	-1.36	0.19
	Item 15	1.59 (0.29)	1.53 (0.29)	-0.50	0.62
	Total	3.47 (0.16)	3.67 (0.16)	2.03	0.06
Module 2 – External Processes –	Item 1	3.56 (0.54)	3.64 (0.54)	0.16	0.88
	Item 2	4.39 (0.50)	3.84 (0.50)	-1.36	0.19
	Item 3	5.12 (0.38)	5.41 (0.38)	1.46	0.16
	Item 4	3.99 (0.49)	4.10 (0.49)	0.28	0.79
	**Item 5**	**3.91 (0.52)**	**4.87 (0.52)**	**2.48**	**0.025**
	Item 6	4.62 (0.50)	4.87 (0.49)	0.81	0.43
	Item 7	3.89 (0.53)	4.27 (0.53)	0.92	0.37
	**Item 8**	**2.26 (0.45)**	**3.31 (0.46)**	**2.49**	**0.024**
	Total	3.97 (0.25)	4.28 (0.25)	1.69	0.11

Legend: bold = significant results; for details about the item nomenclature, please see supplementary material

### Overall preference

Most patients preferred the and blue MEPP 2.0 (OR = 4.57; *p* < 0.001). A chi-square test showed that the preference did not vary according to the order of presentation (χ^2^ = 0.58, df = 1, *p* = 0.44). All clinicians preferred the second version. No statistical analyses can be performed because there are no variances, but the effect is very clear regardless of the order of presentation.

### Qualitative impressions

At the end of each interview, participants were asked about their qualitative impressions of their experiences. For the green MEPP 1.0, the patients generally offered suggestions for improvements primarily related to technology (enhanced facial imaging), restrictive use (requirement to remain centered on the screen) and accessibility (limited to computer use only). Many of these concerns were addressed in the blue MEPP 2.0. Patients often reported the MEPPb2.0 was easier to use, with better image rendering and greater practicality (available on tablets and phones). However, a recurrent comment for the blue MEPP 2.0 was the need for availability on Android devices. Other suggestions varied, with some patients suggesting features such as session recording for future clinician feedback, enhanced exercise support (e.g., demonstration videos), and improved tracking of progress (for instance, before-and-after photos, progress bars during sessions or weekly progress tracking).

Clinicians’ suggestions for improvements in both versions of the MEPP varied and were specifically targeted for better and broader clinical use of the tool. Feedback for the green MEPP 1.0 generally focused on improving information organization (for patients and exercises), ensuring confidentiality (e.g., preventing all clinicians from seeing every patient using the tool) and addressing restrictions on patient usage (limited mobility permitted/having to remain still). Most of these were addressed in the second version and justified the clinicians’ preference for this version. However, further suggestions for improvements included improved exercise categorization or tool personalization (such as exercise banks, favorites, and checkbox options), broader accessibility (e.g., offline use, patient transfer between clinicians), translation of exercises in various languages and the inclusion of a clinician-sharing forum within the application.

## Discussion

The present study aimed to compare the use of the green MEPP 1.0 and blue MEPP 2.0 regarding user experience and patient embodiment as well as to to measure clinical factors affecting their use. Indeed, to avoid the abandonment of rehabilitation technologies, it is crucial to consider users’ opinions on the quality and performance of these technologies [19]. Thus, the use of a user-centered approach aimed at evaluating the usability of the technology by users as well as their perception of the technology’s quality is recommended [8].

Perhaps unsurprisingly, we observed in this study that blue MEPP 2.0 was widely preferred by all users, both clinicians and patients, in terms of user experience. Indeed, all values of the analytical model of user experience developed by Thüring and Mahlke [24] were higher for blue MEPP 2.0. This included the perception of instrumental qualities (usability and utility) and non instrumental qualities (aesthetics, etc.) of the technology, the influence of user emotions on the product, the consequences of these components on loyalty and intention to use the product, and the overall evaluation of the product by the user [14]. These results are promising for the integration and adoption of the tool in clinical settings at large. However, our analysis demonstrated that despite higher values obtained for blue MEPP 2.0, only some showed a statistically significant difference compared to the green MEPP 1.0. This was mostly due to the order of presentation of the two tools. Indeed, when experienced first, the green MEPP 1.0 tended to be better evaluated and gathered more positive feedback than when it was presented after blue MEPP 2.0. These findings are evidenced by statistically non-significant differences between both tools for patients regarding usefulness (“with this product, I achieve my goals”), status (“by using this product, I will be perceived differently”), commitment (“I would not swap this product for another one”), negative emotions toward the green version (“this product bothers me”) or positive emotions toward the blue version (“this product makes me happy”), intention to use (“I cannot hardly wait to use the product again”), and overall evaluation. For clinicians, similar findings were observed with statistically non- significant differences between the tools for negative emotions and intention to use. We hypothesize that when the green MEPP 1.0 is presented first, the absence of experience with a comparable product gives it greater appreciation than when it can be compared to the second version. Furthermore, we believe that users may become accustomed to the initial version used, having a lower propensity to change it, and being less inclined to deviate from it. Indeed, among the factors influencing technology use, researchers have shown that a lack of time to learn [15] or a lack of interest in adopting a new product [25] are significant parameters of influence. Subjective factors of a user, such as personal motivation, perceptions of the product, and emotions, influence the likelihood of using a system and integrating it into everyday life [13].

Regarding virtual embodiment factors, in line with our hypothesis, our results demonstrated that compared with the first version, the blue MEPP 2.0 significantly improved the appropriateness of virtual faces. As highlighted in our previous studies [10], patients and clinicians frequently reported being bothered by the instability of facial mirroring by the central axis. The instability of the mirror effect with this technology results in a less natural face, leading us to hypothesize that this could hinder patients’ ability to appropriate the image reflected by the site. The concept that describes the ability to integrate an image of ourselves as our own body is called embodiment [12]. To remedy the embodiment problem with the central axis, we used augmented reality technology to produce a mirror effect, which allowed for a more realistic and stable rendering of the patients’ faces. Our current results suggest that it promoted embodiment during the rehabilitation process, as reported in previous studies [3, 5, 17]. Importantly, for patients, the visual feedback modified in the blue MEPP 2.0 provides information that is discordant with the intrinsic muscular proprioceptive feedback, which is generally weak due to the absence of neuromuscular bundles in the facial muscles [2]. However, the desired effect is precisely a different and normalized feedback, as it will not only promote the achievement of a more precise and unforced movement but also offer the patient a pleasant-looking image [11]. This observation is motivating, and the result corresponds to the achievement of the desired goals, namely, the realization of symmetrical movements [18]. All these aspects (unforced movement, pleasant image, visual recording of symmetrical movements, achievement of the desired goal) should be taken into consideration in the appreciation of the blue MEPP 2.0. These effects are specifically related to the mirror effect component and point to the added value of the mirror effect as a unique tool for facial rehabilitation. Moreover, for patients with facial paralysis, being visually confronted with facial paralysis through a mirror, for example, during facial exercises, can have detrimental effects on their motivation and self-esteem [23]. Not only has this fact been studied, but it has also been observed within our study through frequent patient comments on this matter. For example, they appreciate working with the mirror effect because they feel like they are returning to how they were before, or they mention that working in front of the mirror gives them real-time and tangible insight into the goals they would like to achieve. It would be interesting to concretely measure this motivational aspect in a comparative manner.

In terms of clinical factors, we expected comparable results for both tools since our participants worked in similar clinical contexts, using two versions of the same therapeutic tool having the same clinical goals. Thus, we thought that the intrinsic characteristics of the technology itself would be more likely to influence the preference for one version over the other, not clinical factors related to the use of the tools. However, our results showed a significant difference in certain factors among clinicians, without an order effect, prompting preference for second version regardless of whether it was used first or second. Clinicians felt that they could gain experience with facial paralysis using the blue MEPP 2.0 and could intervene and assess patient progress with more objectivity. It seems that some improvements made in the blue MEPP 2.0 allowed clinicians to follow patient progress throughout therapy with more accuracy and specificity compared to the previous version. For example, a clinician can see through a visual graph on his or her homepage the number of sessions completed by a patient and even compare the completion of exercise sessions between patients in his or her caseload. Easier follow-up of a patient’s progress and frequency of therapies may facilitate self-assessment in clinical practice, an important aspect in clinical practice to ensure quality care and clinical excellence [7, 16]. Moreover, the preference for blue MEPP 2.0 was also influenced by continuing education, as reported by clinicians. This may be because a video tutorial was available to guide clinicians using the blue MEPP 2.0 and therefore facilitated its use. Finally, the use of the second version was also influenced by information reported by patients. We hypothesize that the use of augmented reality achieves several objectives in patients, including reducing interruptions and extrinsic complexity to therapy related to face positioning and consequently reducing the cognitive load required for the task. Thus, since some patients reported positive feedback to their clinicians concerning the blue MEPP 2.0, clinicians may have been more inclined to prefer it. We also think that the influence of patients’ opinions is particularly interesting because it corroborates the results related to improvements in embodiment among patients. These conclusions are promising for future use since it is suggested that one factor contributing to long-term adoption of a technology is user enjoyment, which supplies motivation to use said technology [15].

### Study limitations

The study is not without limitations. It is important to report that facial paralysis readaptation is an emergent practice in Quebec for speech-language pathologists. Therefore, clinical expertise is limited, and thus, this technology has not been applied only in therapeutic context, but in the context of a research setting. Time constraints also affect technology education and training, especially for clinicians, who are considered important contributors to technology use [15, 25]. Some clinicians using the websites for the first time and for whom technology education was difficult to give had a limited understanding of certain characteristics of the technologies after the given 2-week period. This limitation can therefore affect their appreciation of the two versions of the MEPP and their answers during the interviews. Notably, both questionnaires used in this study (the MeCUE and VEQ) were originally developed in English. Given that Quebec is predominantly French-speaking, both questionnaires had to be freely translated in French by the study team, as there were no validated translations readily available. Finally, we gathered qualitative impressions about the tools, but we did not use any formal qualitative analyses to explore these data.

## Conclusion

In conclusion, the updated version of the website, the blue MEPP 2.0, was more appreciated by both clinicians and patients. The findings suggested that the use of augmented reality technology in the blue MEPP 2.0 was greatly appreciated by patients because it helped them achieve facial stability. Concerning user experience, despite the impact of habit of use, overall, the results showed that blue MEPP 2.0 was a more useful and aesthetic tool and provided more positive emotions to its users. Finally, clinical factors showed the clinician’s preference for the blue MEPP 2.0 on certain aspects of progress assessment, objectivity, experience gain and continuous training. The great appreciation of the blueMEPP 2.0 by clinicians and patients is very promising for the integration and adoption of this technological tool in a clinical setting at large, therefore enhancing facial paralysis rehabilitation in Quebec.

For future directions and studies, we will keep collecting data on the clinical utility of the mirror therapy, to support its use in facial paralysis rehabilitation. Moreover, Focus Group Interviews could be added in future user experience studies, adding to the qualitative aspects of the research.

## Supplementary Information

Supplementary Material 1. Supplementary file 1. Descriptive Statistics for the MeCue subscale by MEPP version (either Green- V1.0 or Blue-V2.0) for all Participants. Supplementary file 2. Patient structure of the interviews and French translation of the two questionnaires. Supplementary file 3. Clinician structure of interviews and French translation of the two questionnaires. Supplementary file 4. Original English version of the Modular Evaluation of Components of User Experience Questionnaire (MeCUE). Supplementary file 5. Original English version of the Virtual Embodiment Questionnaire (VEQ). Supplementary file 6. Datasets.

## Data Availability

The authors confirm that the data supporting the findings of this study are available within the article and its supplementary materials.
