# Supplementary material for: Factors that facilitate or hinder the use of the facial rehabilitation webtool MEPP 2.0: a comparative study in the Quebecer health system
Source: BMC Health Serv Res. 2024 Oct 18;24:1256. doi: 10.1186/s12913-024-11628-2 (PMC11487791; doi:10.1186/s12913-024-11628-2)
Supplement: Supplementary file 1 — Supplementary Material 1. Supplementary file 1. Descriptive Statistics for the MeCue subscale by MEPP version (either Green- V1.0 or Blue-V2.0) for all Participants. Supplementary file 2. Patient structure of the interviews and French translation of the two questionnaires. Supplementary file 3. Clinician structure of interviews and French translation of the two questionnaires. Supplementary file 4. Original English version of the Modular Evaluation of Components of User Experience Questionnaire (MeCUE). Supplementary file 5. Original English version of the Virtual Embodiment Questionnaire (VEQ). Supplementary file 6. Datasets. [file 12913_2024_11628_MOESM1_ESM.docx]

Supplementary file 1 – Original English version of the Modular Evaluation of Components of User Experience Questionnaire (MeCUE)

**Evaluation of interactive products**

***meCUE 2.0* questionnaire**

**
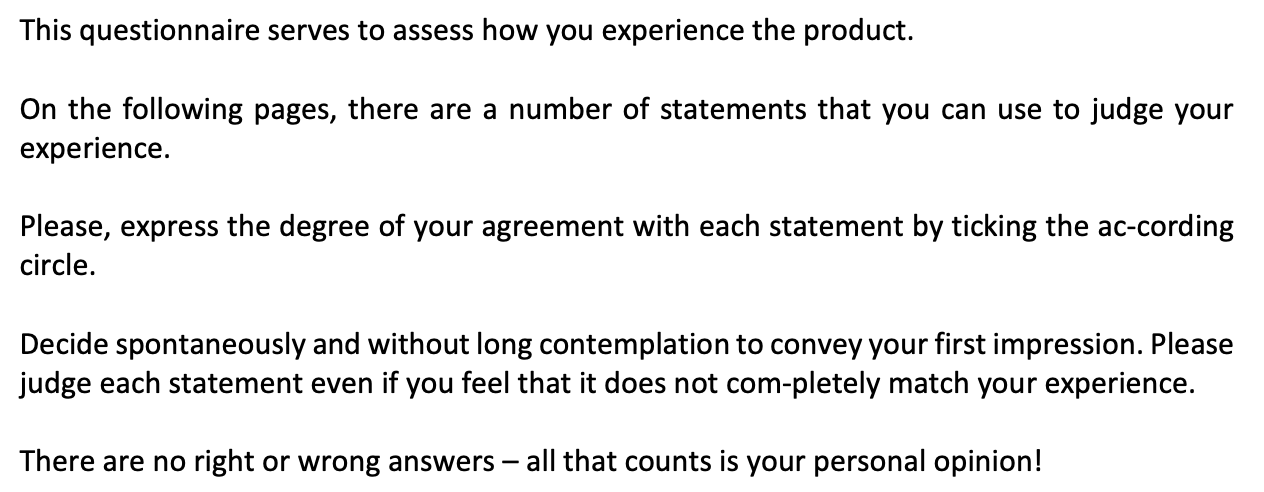
**

**
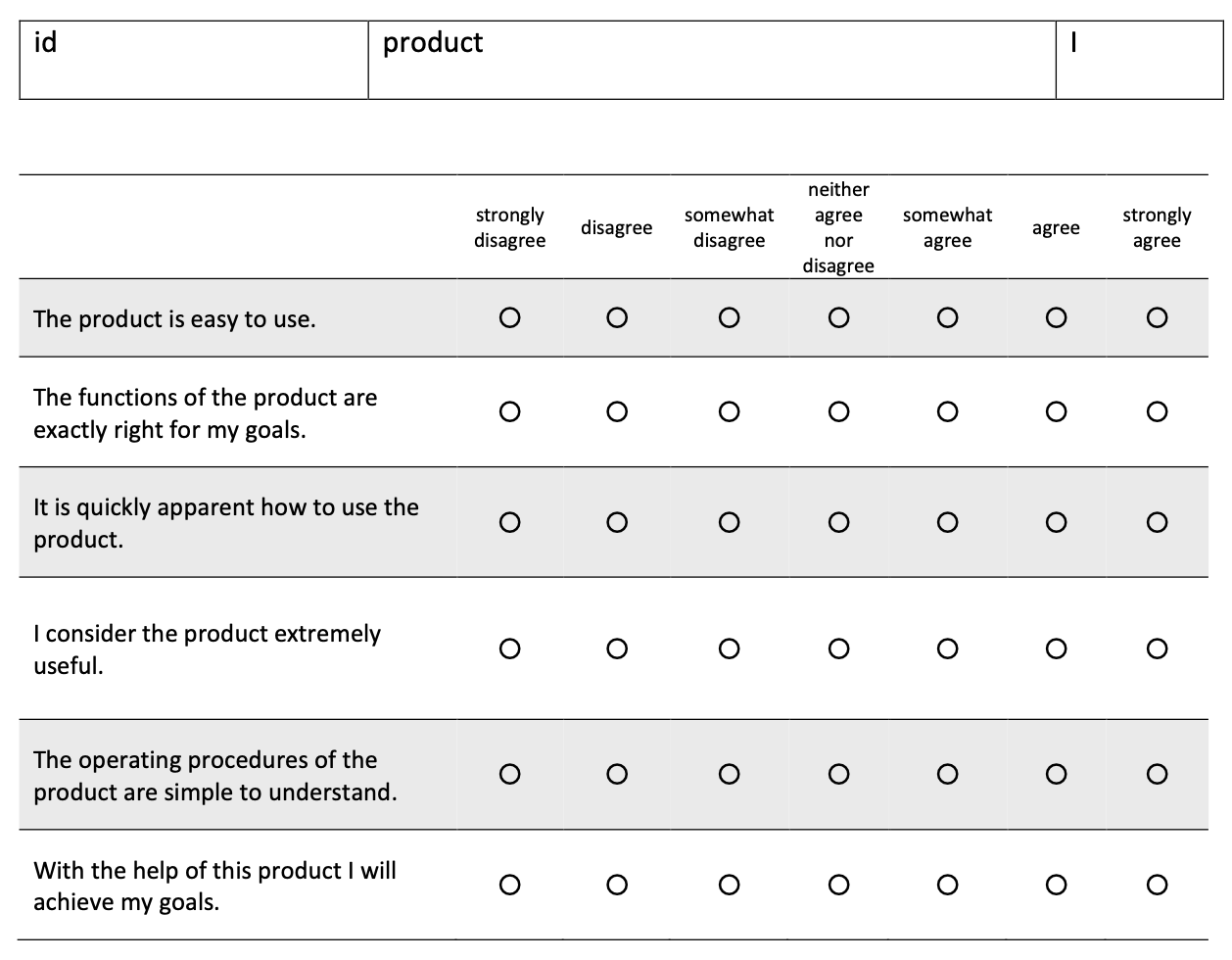
**

**
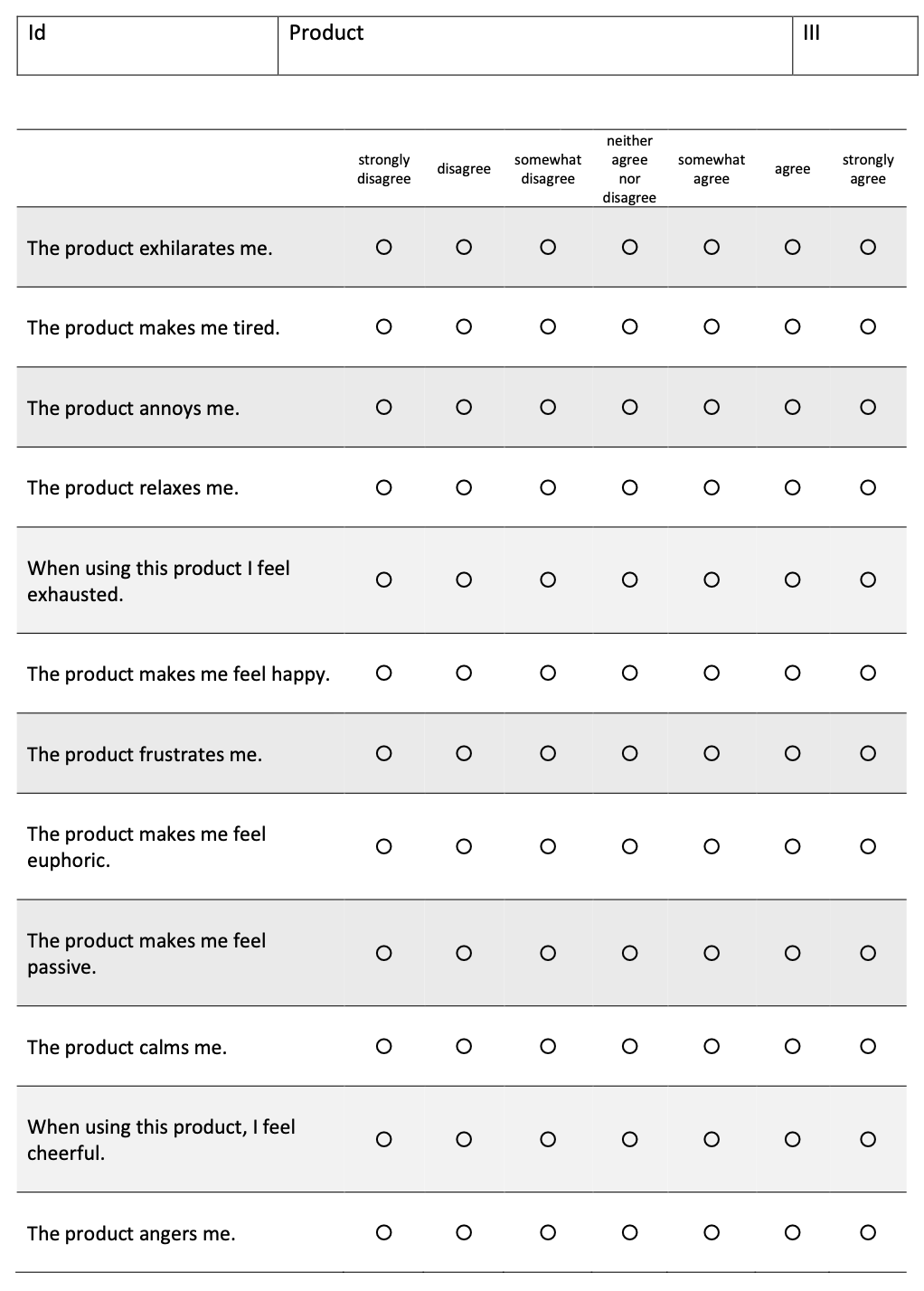
**

**
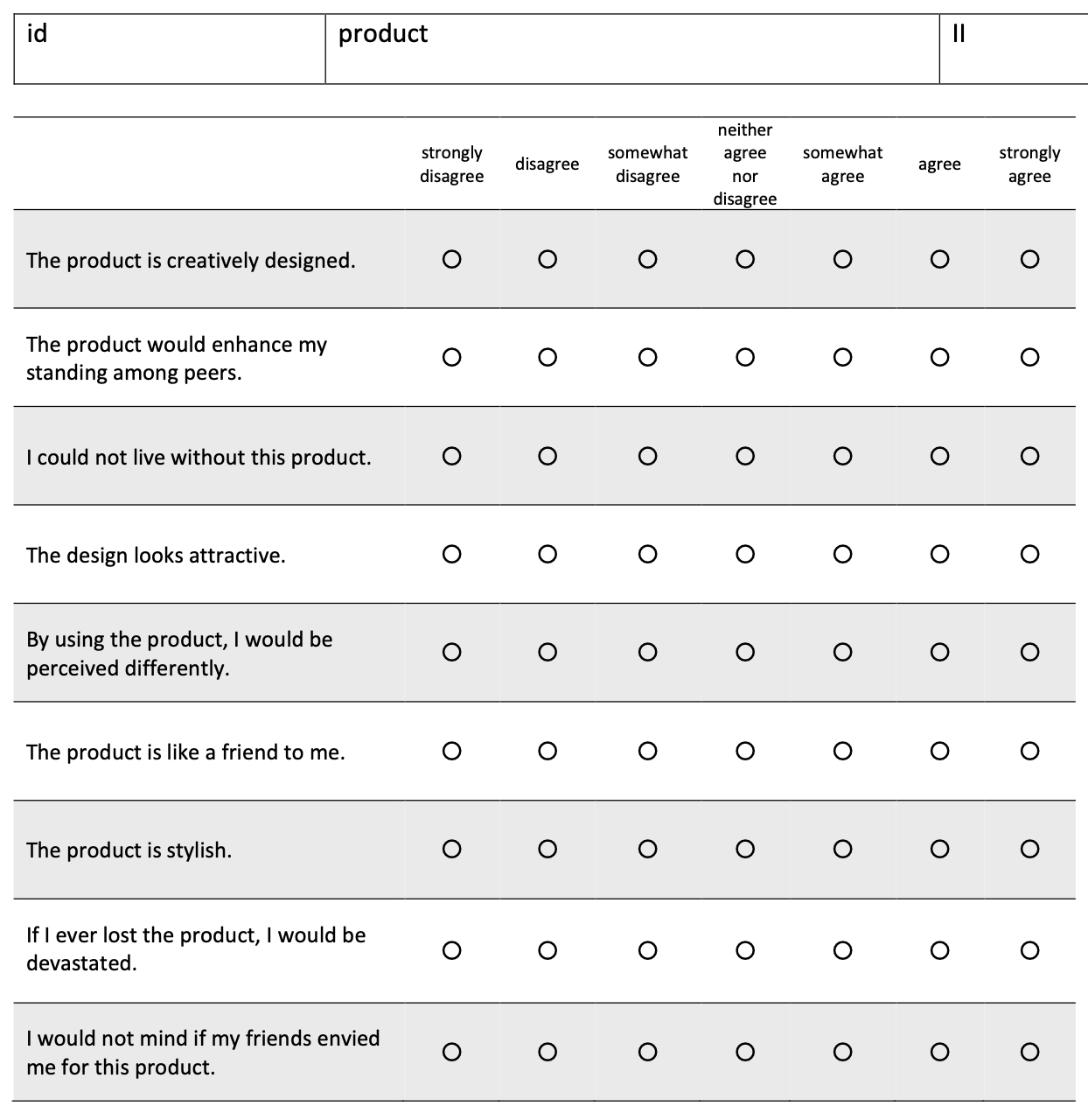
**

**
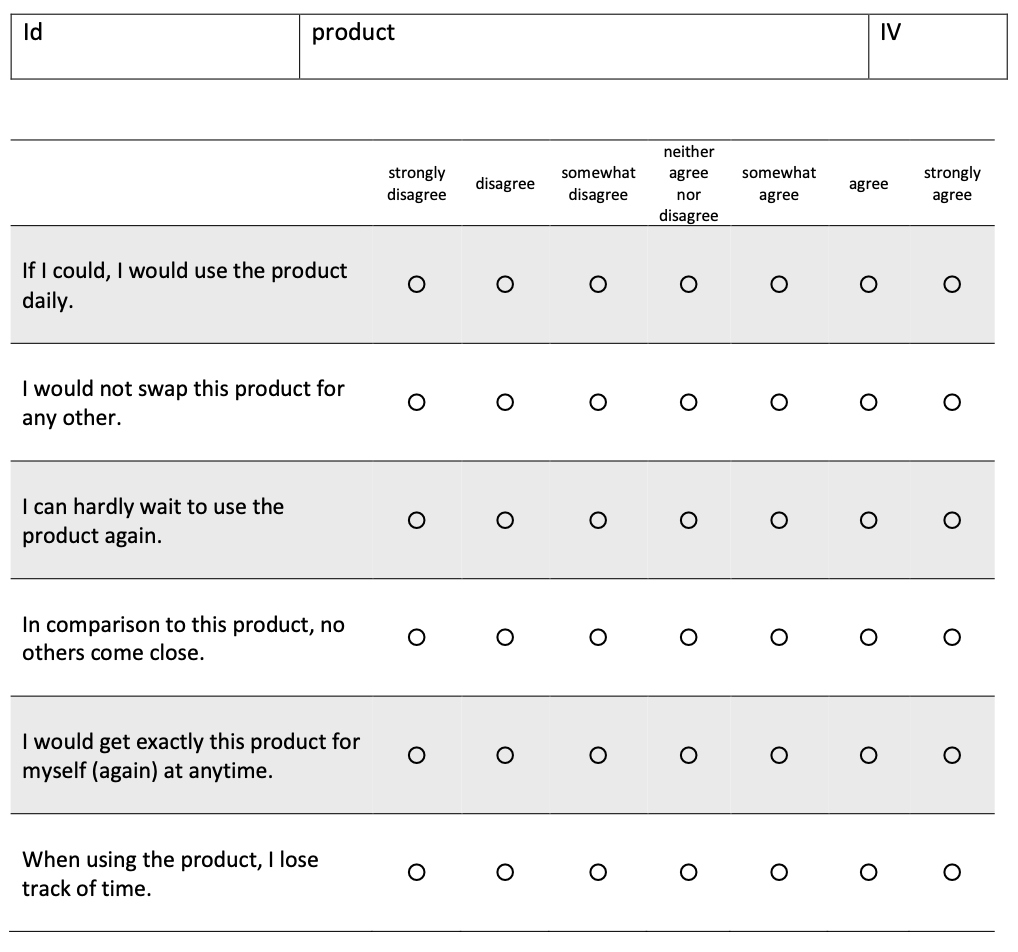
**

**
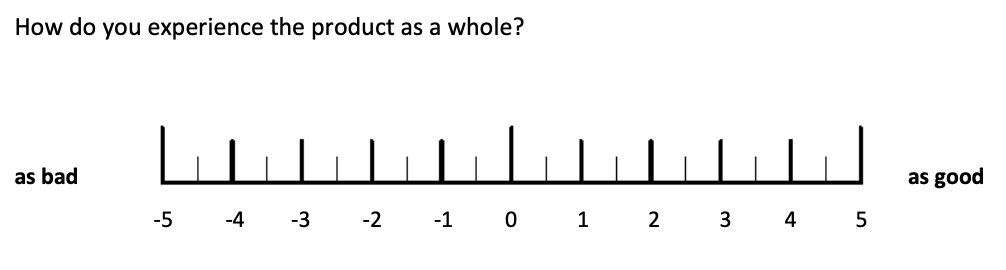
**

Supplementary file 2 – Original English version of the Virtual Embodiment Questionnaire (VEQ)

**Virtual Embodiment Questionnaire (VEQ) - English**

**
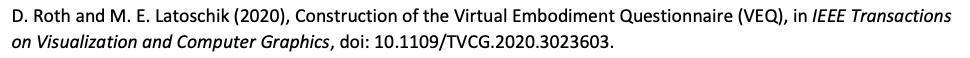
**

**
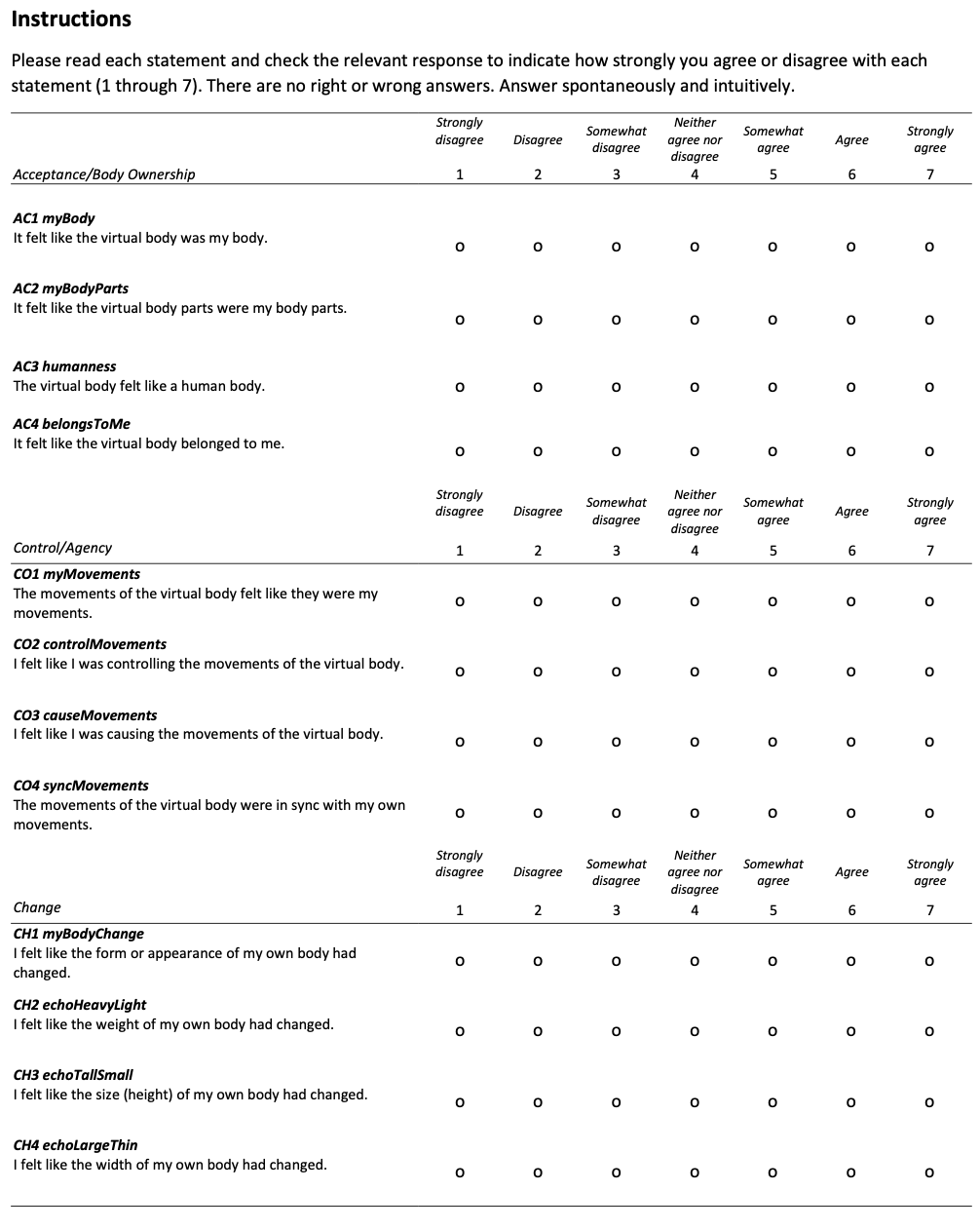
**


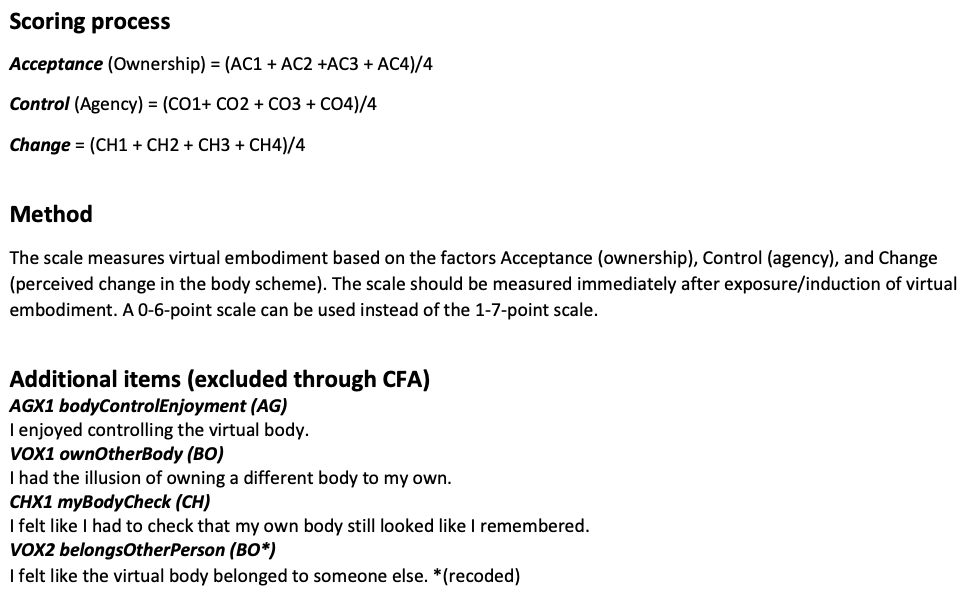


Supplementary file 3 – English version of the Questionnaire assessing factors influencing practice

## SELF-ASSESSMENT ON FACTORS INFLUENCING CLINICAL PRACTICE

## (Based on Orest, 1995)

Instructions**:**

Please read each statement and check your response to indicate your agreement or disagreement with each statement. There are no right or wrong answers. Respond spontaneously and intuitively.

**Module 1. Internal Self-Assessment Processes**

**« THE USE OF THE WEB TOOL…**

|  | Strongly disagree | Disagree | Somewhat disagree | Neutral | Somewhat agree | Agree | Strongly agree | Not applicable |
| --- | --- | --- | --- | --- | --- | --- | --- | --- |
| …has been motivated by my desire/feeling to help patients." | ☐ | ☐ | ☐ | ☐ | ☐ | ☐ | ☐ | ☐ |
| … has been motivated by my desire to reduce my insecurity." | ☐ | ☐ | ☐ | ☐ | ☐ | ☐ | ☐ | ☐ |
| … has been motivated by my desire to comply with regulations." | ☐ | ☐ | ☐ | ☐ | ☐ | ☐ | ☐ | ☐ |
| … has been motivated by my desire to enhance my professional esteem." | ☐ | ☐ | ☐ | ☐ | ☐ | ☐ | ☐ | ☐ |
| … has been recommended by colleagues/peers." | ☐ | ☐ | ☐ | ☐ | ☐ | ☐ | ☐ | ☐ |
| … has allowed me to intervene more objectively." | ☐ | ☐ | ☐ | ☐ | ☐ | ☐ | ☐ | ☐ |
| … has allowed me to objectively track the progress of my patient." | ☐ | ☐ | ☐ | ☐ | ☐ | ☐ | ☐ | ☐ |
| … has allowed me to gain experience." | ☐ | ☐ | ☐ | ☐ | ☐ | ☐ | ☐ | ☐ |
| … has been restricted by lack of time." | ☐ | ☐ | ☐ | ☐ | ☐ | ☐ | ☐ | ☐ |
| … has been restricted by my lack of knowledge in the field." | ☐ | ☐ | ☐ | ☐ | ☐ | ☐ | ☐ | ☐ |
| … has been restricted by my heavy workload." | ☐ | ☐ | ☐ | ☐ | ☐ | ☐ | ☐ | ☐ |
| … has been restricted by the risks associated with its use." | ☐ | ☐ | ☐ | ☐ | ☐ | ☐ | ☐ | ☐ |
| … has been restricted by communication difficulties." | ☐ | ☐ | ☐ | ☐ | ☐ | ☐ | ☐ | ☐ |
| … has been restricted by my state of fatigue." | ☐ | ☐ | ☐ | ☐ | ☐ | ☐ | ☐ | ☐ |
| … has been restricted by associated costs.” | ☐ | ☐ | ☐ | ☐ | ☐ | ☐ | ☐ | ☐ |

**Module 2. External Self-assessment Processes**

"The USE OF THE WEB TOOL...

|  | Strongly disagree | Disagree | Somewhat disagree | Neutral | Somewhat agree | Agree | Strongly agree | Not applicable |
| --- | --- | --- | --- | --- | --- | --- | --- | --- |
| …has been influenced by working alone/in a team with this clientele” | ☐ | ☐ | ☐ | ☐ | ☐ | ☐ | ☐ | ☐ |
| … has been influenced by readings on the subject." | ☐ | ☐ | ☐ | ☐ | ☐ | ☐ | ☐ | ☐ |
| …has been influenced by practice changes on the subject." | ☐ | ☐ | ☐ | ☐ | ☐ | ☐ | ☐ | ☐ |
| … has been influenced by resources allocated for my professional development." | ☐ | ☐ | ☐ | ☐ | ☐ | ☐ | ☐ | ☐ |
| … has been influenced by continuous education." | ☐ | ☐ | ☐ | ☐ | ☐ | ☐ | ☐ | ☐ |
| … has been influenced by the opinion of my peers." | ☐ | ☐ | ☐ | ☐ | ☐ | ☐ | ☐ | ☐ |
| … has been influenced by my initial university education." | ☐ | ☐ | ☐ | ☐ | ☐ | ☐ | ☐ | ☐ |
| … has been influenced by information provided by my patient about this tool." | ☐ | ☐ | ☐ | ☐ | ☐ | ☐ | ☐ | ☐ |

**OPEN QUESTIONS:**

Suggestions to improve the webtool?

In your own words, explain what contributed to the use or non use of the webtool in your practice?

(next page)

(At the end of both trials) In your own words, explain which webtool did you prefer and why

THANKS MERCI!

Supplementary file 4 – Descriptive Statistics for the MeCue subscale by MEPP version (either Green- V1.0 or Blue-V2.0) for all Participants

|  |  | **Patients** | | | | | | **Clinicians** | | | | | |
| --- | --- | --- | --- | --- | --- | --- | --- | --- | --- | --- | --- | --- | --- |
|  |  | Green - 1.0 (n=39) | | | Blue -2.0 (n =41) | | | Green - 1.0 (n=19) | | | Blue -2.0 (n =19) | | |
| Module | Score | Mean | SD | Range | Mean | SD | Range | Mean | SD | Range | Mean | SD | Range |
| I | Usefulness | 4.89 | 1.53 | [1, 7] | 5.70 | 1.22 | [2, 7] | 4,89 | 1,38 | [1, 7] | 6,16 | 0,68 | [5, 7] |
|  | Usability | 4.75 | 1.61 | [1, 7] | 6.62 | 0.56 | [5, 7] | 4,18 | 1,75 | [1, 7] | 5,82 | 1,09 | [3, 7] |
| II | Visual Aesthetic | 4.38 | 1.45 | [1,7] | 5.68 | 0.94 | [3, 7] | 4,05 | 1,30 | [1, 6] | 5,72 | 0,93 | [4, 7] |
|  | Status | 3.51 | 1.24 | [1, 6] | 4.04 | 1.40 | [1, 7] | 3,79 | 1,25 | [2, 6] | 5,16 | 0,73 | [4, 7] |
|  | Commitment | 2.62 | 1.33 | [1, 5] | 3.48 | 1.59 | [1, 7] | 2,68 | 1,23 | [1, 6] | 3,12 | 1,02 | [1, 5] |
| III | Positive emotions | 3.43 | 1.40 | [1, 7] | 4.21 | 1.09 | [2, 6] | 3,37 | 1,10 | [1, 6] | 4,19 | 0,75 | [3, 6] |
|  | Negative emotions | 3.05 | 1.31 | [1, 6] | 2.13 | 0.91 | [1, 5] | 2,61 | 1,24 | [1, 5] | 1,65 | 0,76 | [1, 4] |
| IV | Intention to use | 3.58 | 1.47 | [1, 7] | 4.33 | 1.37 | [1, 7] | 3,47 | 1,24 | [1, 6] | 4,39 | 0,79 | [3, 6] |
|  | Product Loyalty | 3.10 | 1.64 | [1, 6] | 5.05 | 1.32 | [1, 7] | 2,89 | 1,30 | [1, 6] | 5,38 | 0,85 | [4, 7] |
| V | Overall | 1.07 | 2.82 | [-5, 5] | 3.45 | 1.61 | [-3, 5] | 0,84 | 2,65 | [-5, 4] | 3,76 | 0,79 | [2, 5] |

Legend: Module I & II: Product Perception; Module III: Emotions; Module IV: Consequences of use; Module V: Overall evaluation

Supplementary file 5: Datasets

MeCUE GREEN MEPP Patients

MeCUE BLUE MEPP Patients

VEQ Patients

| ID NUMBER | MEPP | Module 1 | C’était comme si le visage virtuel était mon corps. | C’était comme si les parties de mon visage virtuelles étaient les parties de mon visage. | C’était comme si le visage virtuel était un visage humain. | C’était comme si le visage virtuel m’appartenait. | Module 2 | C’était comme si les mouvements du visage virtuel étaient mes mouvements. | J’avais l’impression de contrôler les mouvements du visage virtuel. | J’avais l’impression de causer les mouvements du visage virtuel. | Les mouvements du visage virtuel était synchronisé avec mes propres mouvements. | Module 3 | J’avais l’impression que la forme ou l’apparence de mon propre visage avait changé. | J’avais l’impression que la masse de mon propre visage avait changé. | J’avais l’impression que la hauteur de mon propre visage avait changé. | J’avais l’impression que la largeur de mon propre visage avait changé. |
| --- | --- | --- | --- | --- | --- | --- | --- | --- | --- | --- | --- | --- | --- | --- | --- | --- |
| 181BA | 2. Blue |  | 6 | 6 | 6 | 6 |  | 3 | 5 | 5 | 4 |  | 5 | 2 | 1 | 5 |
| 181BA | 1. Green |  | 3 | 4 | 3 | 4 |  | 3 | 5 | 5 | 3 |  | 5 | 2 | 2 | 2 |
| 182AB | 1. Green |  | 5 | 5 | 3 | 5 |  | 5 | 5 | 5 | 5 |  | 6 | 6 | 4 | 5 |
| 182AB | 2. Blue |  | 6 | 6 | 5 | 6 |  | 6 | 7 | 7 | 7 |  | 7 | 5 | 4 | 4 |
| 183AB | 1. Green |  | 1 | 2 | 1 | 1 |  | 3 | 5 | 6 | 6 |  | 6 | 7 | 7 | 7 |
| 183AB | 2. Blue |  | 3 | 3 | 3 | 3 |  | 6 | 1 | 6 | 7 |  | 1 | 1 | 1 | 1 |
| 184BA | 2. Blue |  | 6 | 6 | 6 | 6 |  | 6 | 7 | 7 | 7 |  | 5 | 2 | 2 | 5 |
| 184BA | 1. Green |  | 5 | 6 | 5 | 5 |  | 6 | 6 | 6 | 6 |  | 5 | 5 | 3 | 3 |
| 185AB | 1. Green |  | 1 | 2 | 2 | 2 |  | 1 | 1 | 2 | 1 |  | 7 | 7 | 3 | 7 |
| 185AB | 2. Blue |  | 7 | 5 | 6 | 7 |  | 5 | 5 | 7 | 5 |  |  | 6 | 2 | 6 |
| 186BA | 2. Blue |  | 6 | 6 | 6 | 6 |  | 5 | 5 | 6 | 6 |  | 3 | 2 | 2 | 2 |
| 186BA | 1. Green |  | 4 | 2 | 4 | 4 |  | 4 | 5 | 6 | 6 |  | 4 | 6 | 2 | 6 |
| 187BA | 2. Blue |  | 5 | 5 | 7 | 6 |  | 7 | 7 | 7 | 7 |  | 5 | 2 | 2 | 2 |
| 187BA | 1. Green |  | 2 | 1 | 3 | 1 |  | 3 | 3 | 5 | 6 |  | 5 | 5 | 2 | 7 |
| 188AB | 1. Green |  | 5 | 6 | 5 | 5 |  | 6 | 5 | 5 | 6 |  | 6 | 2 | 2 | 2 |
| 188AB | 2. Blue |  | 2 | 2 | 2 | 2 |  | 3 | 4 | 5 | 6 |  | 6 | 2 | 2 | 2 |
| 241BA | 2. Blue |  | 6 | 7 | 7 | 7 |  | 7 | 7 | 6 | 7 |  | 5 | 2 | 2 | 2 |
| 241BA | 1. Green |  | 2 | 3 | 3 | 2 |  | 7 | 7 | 7 | 3 |  | 7 | 6 | 6 | 7 |
| 242AB | 1. Green |  | 2 | 2 | 2 | 2 |  | 3 | 3 | 5 | 6 |  | 6 | 7 | 7 | 7 |
| 242AB | 2. Blue |  | 2 | 2 | 2 | 2 |  | 5 | 3 | 3 | 3 |  | 5 | 5 | 5 | 5 |
| 243BA | 2. Blue |  | 6 | 6 | 6 | 6 |  | 6 | 6 | 6 | 6 |  | 2 | 2 | 2 | 2 |
| 243BA | 1. Green |  | 7 | 7 | 7 | 7 |  | 7 | 7 | 7 | 7 |  | 1 | 4 | 1 | 6 |
| 244AB | 1. Green |  | 6 | 6 | 6 | 4 |  | 6 | 7 | 7 | 7 |  | 7 | 2 | 1 | 1 |
| 244AB | 2. Blue |  | 5 | 7 | 7 | 7 |  | 6 | 7 | 7 | 7 |  | 7 | 7 | 2 | 2 |
| 341AB | 1. Green |  | 6 | 6 | 6 | 6 |  | 5 | 4 | 4 | 4 |  | 2 | 2 | 2 | 4 |
| 341AB | 2. Blue |  | 2 | 2 | 2 | 2 |  | 2 | 2 | 2 | 3 |  | 6 | 2 | 2 | 2 |
| 342BA | 2. Blue |  | 7 | 7 | 7 | 7 |  | 7 | 7 | 7 | 7 |  | 7 | 7 | 3 | 7 |
| 342BA | 1. Green |  | 5 | 6 | 7 | 7 |  | 7 | 7 | 7 | 7 |  | 7 | 7 | 6 | 7 |
| 343BA | 2. Blue |  | 6 | 6 | 5 | 5 |  | 6 | 6 | 6 | 6 |  | 5 | 5 | 3 | 5 |
| 343BA | 1. Green |  | 1 | 1 | 1 | 1 |  | 6 | 5 | 5 | 6 |  | 7 | 7 | 7 | 7 |
| 344AB | 1. Green |  | 6 | 6 | 6 | 6 |  | 6 | 6 | 6 | 6 |  | 2 | 2 | 2 | 2 |
| 344AB | 2. Blue |  | 6 | 6 | 6 | 6 |  | 6 | 6 | 6 | 6 |  | 2 | 2 | 2 | 2 |
| 461AB | 1. Green |  | 6 | 7 | 6 | 7 |  | 7 | 7 | 7 | 7 |  | 6 | 5 | 6 | 5 |
| 461AB | 2. Blue |  | 5 | 5 | 5 | 5 |  | 7 | 7 | 7 | 7 |  | 3 | 2 | 2 | 2 |
| 462BA | 2. Blue |  | 5 | 5 | 5 | 5 |  | 5 | 5 | 6 | 5 |  | 5 | 3 | 2 | 2 |
| 462BA | 1. Green |  | 3 | 4 | 5 | 2 |  | 5 | 5 | 5 | 5 |  | 5 | 5 | 5 | 5 |
| 463AB | 1. Green |  | 2 | 2 | 5 | 2 |  | 6 | 6 | 6 | 5 |  | 6 | 6 | 2 | 6 |
| 463AB | 2. Blue |  | 6 | 6 | 6 | 6 |  | 6 | 6 | 6 | 6 |  | 2 | 2 | 2 | 2 |
| 464AB | 1. Green |  | 1 | 1 | 2 | 2 |  | 1 | 2 | 6 | 6 |  | 7 | 6 | 4 | 7 |
| 464AB | 2. Blue |  | 4 | 4 | 6 | 6 |  | 6 | 6 | 6 | 5 |  | 6 | 5 | 4 | 2 |
| 465BA | 2. Blue |  | 2 | 2 | 7 | 3 |  | 7 | 7 | 7 | 7 |  | 7 | 1 | 1 | 1 |
| 465BA | 1. Green |  | 1 | 1 | 1 | 1 |  | 5 | 7 | 7 | 7 |  | 6 | 6 | 2 | 6 |
| 466BA | 2. Blue |  | 7 | 7 | 7 | 7 |  | 6 | 0 | 0 | 7 |  | 6 | 2 | 2 | 2 |
| 466BA | 1. Green |  | 0 | 0 | 0 | 0 |  | 0 | 0 | 0 | 0 |  | 0 | 0 | 0 | 0 |
| 541AB | 1. Green |  | 5 | 5 | 6 | 6 |  | 6 | 6 | 6 | 7 |  | 3 | 2 | 2 | 2 |
| 541AB | 2. Blue |  | 2 | 2 | 6 | 6 |  | 6 | 6 | 6 | 6 |  | 7 | 7 | 2 | 7 |
| 542AB | 1. Green |  | 3 | 3 | 4 | 4 |  | 6 | 7 | 6 | 7 |  | 7 | 6 | 7 | 7 |
| 542AB | 2. Blue |  | 5 | 5 | 6 | 5 |  | 3 | 5 | 5 | 6 |  | 6 | 5 | 1 | 5 |
| 543BA | 2. Blue |  | 5 | 5 | 3 | 3 |  | 5 | 3 | 3 | 6 |  | 7 | 2 | 2 | 5 |
| 543BA | 1. Green |  | 1 | 1 | 1 | 3 |  | 6 | 6 | 6 | 6 |  | 5 | 2 | 2 | 4 |
| 544BA | 2. Blue |  | 7 | 7 | 7 | 7 |  | 5 | 5 | 5 | 3 |  | 1 | 1 | 1 | 1 |
| 544BA | 1. Green |  | 1 | 2 | 4 | 4 |  | 2 | 3 | 3 | 3 |  | 7 | 7 | 3 | 3 |
| 622BA | 2. Blue |  | 6 | 5 | 6 | 6 |  | 5 | 7 | 6 | 7 |  | 6 | 5 | 3 |  |
| 622BA | 1. Green |  | 1 | 1 | 5 | 2 |  | 5 | 5 | 5 | 5 |  | 6 | 6 | 3 | 6 |
| 661BA | 2. Blue |  | 7 | 7 | 7 | 7 |  | 7 | 7 | 7 | 7 |  | 6 | 1 | 1 | 6 |
| 661BA | 1. Green |  | 1 | 5 | 4 | 1 |  | 5 | 2 | 5 | 5 |  | 6 | 6 | 2 | 6 |
| 663AB | 1. Green |  | 5 | 5 | 3 | 3 |  | 3 | 5 | 5 | 1 |  | 6 | 5 | 5 | 5 |
| 663AB | 2. Blue |  | 3 | 3 | 3 | 3 |  | 5 | 5 | 6 | 6 |  | 5 | 5 | 2 | 5 |
| 664BA | 2. Blue |  | 6 | 7 | 6 | 7 |  | 7 | 7 | 7 | 7 |  | 2 | 2 | 2 | 2 |
| 664BA | 1. Green |  | 3 | 5 | 6 | 7 |  | 7 | 7 | 7 | 7 |  | 6 | 7 | 3 | 5 |
| 665AB | 1. Green |  | 5 | 5 | 5 | 5 |  | 6 | 6 | 6 | 6 |  | 3 | 3 | 3 | 3 |
| 665AB | 2. Blue |  | 6 | 6 | 6 | 6 |  | 6 | 6 | 6 | 6 |  | 2 | 2 | 2 | 2 |
| 666AB | 1. Green |  |  |  |  |  |  |  |  |  |  |  |  |  |  |  |
| 666AB | 2. Blue |  | 6 | 6 | 6 | 6 |  | 6 | 6 | 6 | 6 |  | 5 | 6 | 5 | 5 |
| 741AB | 1. Green |  | 6 | 6 | 6 | 6 |  | 6 | 6 | 6 | 6 |  | 2 | 6 | 2 | 6 |
| 741AB | 2. Blue |  | 5 | 6 | 6 | 6 |  | 6 | 6 | 6 | 5 |  | 5 | 5 | 2 | 5 |
| 742AB | 1. Green |  | 1 | 2 | 2 | 1 |  | 7 | 7 | 7 | 7 |  | 7 | 7 | 7 | 7 |
| 742AB | 2. Blue |  | 7 | 7 | 7 | 7 |  | 7 | 7 | 6 | 7 |  | 2 | 2 | 1 | 1 |
| 743BA | 2. Blue |  | 5 | 5 | 6 | 6 |  | 6 | 6 | 6 | 5 |  | 2 | 2 | 2 | 2 |
| 743BA | 1. Green |  |  |  |  |  |  |  |  |  |  |  |  |  |  |  |
| 881BA | 2. Blue |  | 5 | 5 | 6 | 5 |  | 6 | 6 | 7 | 7 |  | 5 | 2 | 1 | 4 |
| 881BA | 1. Green |  | 1 | 1 | 1 | 1 |  | 1 | 2 | 2 | 3 |  | 7 | 7 | 4 | 7 |
| 882BA | 2. Blue |  | 6 | 6 | 6 | 6 |  | 6 | 6 | 6 | 6 |  | 2 | 5 | 2 | 5 |
| 882BA | 1. Green |  | 3 | 4 | 4 | 4 |  | 4 | 4 | 4 | 4 |  | 4 | 4 | 4 | 4 |
| 883BA | 2. Blue |  | 5 | 5 | 5 | 5 |  | 6 | 5 | 6 | 5 |  | 2 | 2 | 2 | 2 |
| 883BA | 1. Green |  | 5 | 5 | 6 | 6 |  | 6 | 6 | 3 | 6 |  | 2 | 2 | 2 | 2 |
| 884BA | 2. Blue |  | 1 | 5 | 7 | 7 |  | 5 | 5 | 5 | 5 |  | 5 | 2 | 2 | 2 |
| 884BA | 1. Green |  |  |  |  |  |  |  |  |  |  |  |  |  |  |  |
| 885AB | 1. Green |  | 6 | 6 | 6 | 6 |  | 6 | 5 | 6 | 6 |  | 2 | 2 | 3 | 3 |
| 885AB | 2. Blue |  | 6 | 6 | 6 | 6 |  | 6 | 5 | 6 | 6 |  | 2 | 2 | 2 | 2 |
| 886AB | 1. Green |  | 2 | 2 | 2 | 2 |  | 2 | 4 | 6 | 6 |  | 6 | 6 | 6 | 6 |
| 886AB | 2. Blue |  | 6 | 6 | 6 | 6 |  | 6 | 6 | 6 | 6 |  | 6 | 6 | 6 | 6 |

Patients’preference

| ID NUMBER | Favorite MEPP |
| --- | --- |
| **181BA** | 2. Blue |
| **182AB** | 2. Blue |
| **183AB** | 2. Blue |
| **184BA** | 2. Blue |
| **185AB** | 2. Blue |
| **186BA** | 2. Blue |
| **187BA** | 2. Blue |
| **188AB** | 1. Green |
| **241BA** | 2. Blue |
| **242AB** | 1. Green |
| **243BA** | 1. Green |
| **244AB** | 2. Blue |
| **341AB** | 1. Green |
| **342BA** | 2. Blue |
| **343BA** | 2. Blue |
| **344AB** | 2. Blue |
| **461AB** | 2. Blue |
| **462BA** | 2. Blue |
| **463AB** | 2. Blue |
| **464AB** | 2. Blue |
| **465BA** | 2. Blue |
| **466BA** | 2. Blue |
| **541AB** | 1. Green |
| **542AB** | 2. Blue |
| **543BA** | 2. Blue |
| **544BA** | 2. Blue |
| **622BA** | 2. Blue |
| **661BA** | 2. Blue |
| **663AB** | 2. Blue |
| **664BA** | 1. Green |
| **665AB** | 2. Blue |
| **666AB** | 2. Blue |
| **741AB** | 1. Green |
| **742AB** | 2. Blue |
| **743BA** |  |
| **881BA** | 2. Blue |
| **882BA** | 2. Blue |
| **883BA** | 2. Blue |
| **884AB** |  |
| **885AB** | 2. Blue |
| **886AB** | 2. Blue |

MeCUE GREEN MEPP Clinicians

MeCUE BLUE MEPP Clinicians

Clinical factors Clinicians

Clinicians’ preference

| ID NUMBER | Favorite MEPP |
| --- | --- |
| 181BA | 2. Blue |
| 182AB | 2. Blue |
| 183AB | 2. Blue |
| 184BA | 2. Blue |
| 185AB | 2. Blue |
| 186BA | 2. Blue |
| 187BA | 2. Blue |
| 188AB | 2. Blue |
| 241BA | 2. Blue |
| 242AB | 2. Blue |
| 243BA | 2. Blue |
| 244AB | 2. Blue |
| 341AB | 2. Blue |
| 342BA | 2. Blue |
| 343BA | 2. Blue |
| 344AB | 2. Blue |
| 482AB | 2. Blue |
| 483AB | 2. Blue |
| 484AB | 2. Blue |
|  |  |
